# Appreciation Is Sweet

**Published:** 1913-06

**Authors:** J. M. Massay

**Affiliations:** 4301 South Wesley Street; Greenville, Texas


### Appreciation is Sweet.

GREENVILLE, TEXAS, May 29, 1913.

*Mrs. Josephine Daniel, Woman's Department of the "Red Back," Austin, Texas.*

DEAR MRS. DANIEL: I admire the "Red Back" because it teaches men and women the divinity of motherhood and fatherhood. The mothers and fathers should know the inevitable will come to cloud the home and weaken and ruin immortal life if we are not clean. The "Red Back" teaches the scientific facts in regard to alcohol on the human system. The Woman's Department is very fine, as it tells us the truth on vital subjects so necessary to the moulding of character. It upholds the single standard of morals that we *must* have. The "Red Back" coincides with *Good Health*, a clean journal condemning the use of alcohol as a medicine. After studying "Alcohol a Dangerous and Unnecessary Medicine," by Mrs. Martha M. Allen, we know better how to appreciate journals that teach the truth in regard to narcotics. We as a local union will help to place the "Red Back" in many homes, as we know it will make us wiser and educate mothers, and when we educate a mother we lift the nation higher. Yours in the work.

(Mrs.) J. M. Massay.

4301 South Wesley Street.

---

The above is not all. At the recent State convention in San Antonio when the subject of the "Red Back" was introduced, Mrs. Eaton of Tyler said that in addition to using it in the Union they placed it in the public library. Mrs. T. S. Clark, recording secretary, was most enthusiastic in her commendation of the "Red Back" and especially of its Woman's Department and its editor. Miss Fannie Armstrong of Fort Worth said she was "nothing but an old maid," didn't know as much as some married folks, but she had had to study and learn a great many things, because of the mortal ignorance of so many mothers, so she could help them, and tell them things. She said she was doing all she could to advance the interest of the Texas Medical Journal. Mrs. J. B. Ammerman of San Benito, said her Union had been using the "Red Back" in their Mothers' Meetings, and found it simply invaluable. She said they had a number of young women with them, and was so glad that they could hear the subjects in the presence of the mothers. And so the good work goes on. Such testimonials are most grateful and cheering and we return our thanks.

